# Predicting ICU survival: A meta-level approach

**DOI:** 10.1186/1472-6963-8-157

**Published:** 2008-07-26

**Authors:** Lefteris G Gortzis, Filippos Sakellaropoulos, Ioannis Ilias, Konstantinos Stamoulis, Ioanna Dimopoulou

**Affiliations:** 1 Telemedicine Unit, School of Medicine, University of Patras, Patras, Greece; 2 Department of Endocrinology, Elena Venizelou Hospital, Athens, Greece; 3 Second Department of Critical Care Medicine, Attikon Hospital, Medical School, University of Athens, Athens, Greece

## Abstract

**Background:**

The performance of separate Intensive Care Unit (ICU) status scoring systems vis-à-vis prediction of outcome is not satisfactory. Computer-based predictive modeling techniques may yield good results but their performance has seldom been extensively compared to that of other mature or emerging predictive models. The objective of the present study was twofold: to propose a prototype meta-level predicting approach concerning Intensive Care Unit (ICU) survival and to evaluate the effectiveness of typical mining models in this context.

**Methods:**

Data on 158 men and 46 women, were used retrospectively (75% of the patients survived). We used Glasgow Coma Scale (GCS), Acute Physiology And Chronic Health Evaluation II (APACHE II), Sequential Organ Failure Assessment (SOFA) and Injury Severity Score (ISS) values to structure a decision tree (DTM), a neural network (NNM) and a logistic regression (LRM) model and we evaluated the assessment indicators implementing Receiver Operating Characteristics (ROC) plot analysis.

**Results:**

Our findings indicate that regarding the assessment of indicators' capacity there are specific discrete limits that should be taken into account. The Az score ± SE was 0.8773± 0.0376 for the DTM, 0.8061± 0.0427 for the NNM and 0.8204± 0.0376 for the LRM, suggesting that the proposed DTM achieved a near optimal Az score.

**Conclusion:**

The predicting processes of ICU survival may go "one step forward", by using classic composite assessment indicators as variables.

## Background

Recent research has focused on the appropriateness of care and on the clinical performance of Intensive Care Unit (ICU) hospitalization. This quest for a measure of ICU performance has led to the development of severity adjustment systems that will allow standardized comparisons of outcome and resource use across ICUs [1]. These systems, for many years used only in research settings, have evolved to become sophisticated, computer-based decision-support tools, in some instances commercially developed, and capable of predicting a diverse set of outcomes

Consensus review by experts in critical care or the implementation of models such as multivariate linear and logistic regression have led to the creation of scoring systems such as the Acute Physiology And Chronic Health Evaluation (APACHE) or Sequential Organ Failure Assessment (SOFA). These scoring tools include physiological variables (such as heart or respiratory rate); the overall score follows the rule that higher scores represent more severe illness [1-3].

Until now, nevertheless, the performance of each of them separately vis-à-vis prediction of outcome has not been satisfactory [1]. Regardless of advances in their conception and implementation their predictive potential for survival is usually not better than that of clinical judgment (for example the APACHE II score is more predictive in case of low, < 30%, mortality, whereas overall its Receiver Operating Characteristics (ROC) Area Under the Curve (AUC) is 39%–85% vis-à-vis a 51%–85% ROC AUC for clinical judgment in predicting survival)[4] Even external validation performed on thousands of ICU admissions yields only slightly worse ROC AUCs for tools such as the APACHE II, Simplified Acute Physiology Score (SAPS II), Mortality Probability Model on ICU admission (MPM_0_-II) or Mortality Probability Model 24 hours after ICU admission (MPM_24 _II) compared to initial studies that introduced them to clinical practice [5]. Furthermore, it should be noted that although ICU outcome predicting systems are very important statistical tools for quality management or clinical trials (i.e. in implementing stratification or for clustering) in several cases their potential concerning judgment about an individual therapy may be limited.

Several approaches are used for audit purposes, and some are used as performance measures and quality indicators of a unit; however, both utilities are controversial because of poor adjustment of these systems to case-mixtures [6]. From a review of several studies, although discrimination between survivors and non-survivors was shown to be fair to good, calibration was insufficient in most studies [7]. Furthermore, general prognostic models uniformly underestimate the likelihood of hospital mortality [7]. Neural networks models (NNMs) and other computer-based predictive modeling techniques e.g. decision tree models (DTMs), have yielded better results than logistic regression models (LRMs), or assessment tools such as the APACHE or the Injury Severity Score (ISS) alone [8-10]. Despite increased interest in NNMs for prognosis, their performance has seldom been extensively compared to that of other mature or emerging predictive models [11]. Therefore a combination of stationary and non-stationary models may enable better prediction outcomes [12]. However, usually deficiencies in design, methodology, and reporting make interpretation and combination difficult [13].

The objective of the present study was twofold: to propose a prototype meta-level predicting approach concerning Intensive Care Unit (ICU) survival and to evaluate the effectiveness of using typical mining models in this context using ROC plot analysis.

## Methods

### Subjects

This was a retrospective study of 158 men and 46 women (mean age 49.7± 20.3 years), patients hospitalized at a single tertiary-care teaching hospital's ICU from August 2003 to December 2005. Ninety-four patients suffered from severe trauma. Thus, trauma patients made up approximately 46% of patients. Of the remaining patients 19% were admitted to the ICU for pulmonary disease (pulmonary embolism, infections, respiratory failure), 16.5% after emergency thoracic or abdominal surgery, 12% for stroke or cerebral hemorrhage, 3% after surgery for neoplastic disease, 1% after cardiac arrest, 1% for other neurological diseases, and 1.5% for drug overdose, burns or drowning. Seventeen percent of patients were transferred from another institution. Median ICU stay was 9 days (Q25:4.5 and Q75:21 days), whereas patients were on mechanical ventilation for a median of 5 days (Q25:2.5 and Q75: 12.5 days). Overall ICU survival rate was 75% (at present we have no data on hand for hospital mortality). The hospital's Clinical/Medical Ethics committee (that oversees intramural clinical as well as research activity) gave permission for performing the study. Informed consent was obtained from the patients' next of kin.

### Measures

Upon admission in the ICU severity of the patient's condition was assessed by means of the Glasgow Coma Scale (GCS; mean± SD: 10.94± 3.86, median 12), the APACHE II score (mean± SD: 11.31+5.18, median: 11), the admission SOFA score (mean+SD: 6.35+2.91, median 6) and the ISS score (mean+SD: 40.13+13.88, median: 38; an ISS score was given only in case of trauma; the latter was applicable and available for the 94 patients with trauma only).

### Study protocol

First, we compared the GCS, ISS, APACHE II, and SOFA between survivors and non-survivors using Student's t-test (for normally distributed data) and the Kruskall-Wallis (K-W) test (for data that were not distributed normally).

A meta-level approach was adopted. The clinical variables GCS, ISS, APACHE, and SOFA were used as the input of a prototype predicting approach concerning ICU survival. In this approach ICU Survival was studied as the predictable entity.

By using the Business Intelligence Development Studio (BI Dev Studio; integrated into Microsoft Visual Studio; Microsoft, Seattle, WA, USA) we structured three typical mining models – a DTM, a NNM, and a LRM. Then, we used k-fold cross validation since, generally, is far more efficient than splitting the sample into training and testing sets and is therefore preferred. Due to our sample size we chose k = 4. The data set was randomly split into 4 mutually exclusive folds D1, D2, D3, D4 of approximately equal size. For each division of the sample the model was developed from the training data (141 samples) and tested in the rest (62 samples). Selection of the subjects was into training and testing groups was made randomly. Then ROC analysis was used to investigate the models' effectiveness. Prediction queries in the structure were defined to join the models with the test set. The Object Linking and Embedding, Database, (OLE DB) for data mining Application-Programming Interface (API)[14] was used to write text-based files (according to ROCKIT input format specifications) and to embed the appropriate data mining components (e.g. schema rowsets, prediction features etc.). Goodness of fit was assed with Hosmer's and Lemeshow's statistic [15].

### Patient management

As mentioned above the potential of outcome predicting systems concerning the judgment about an individual therapy may be limited. Hence, the final judgment was made by the dedicated physician according to the interpretation of the patients' physiological parameters and with a working knowledge of the physiological, ethical and personal aspects of patients.

### ROC analysis

ROC curve analysis provides a description of variable detectability that is independent from both prevalence and decision threshold effects [16]. ROC curves of the models were constructed by plotting the "Sensitivity" (true-positive fraction) on the ordinate as a function of the complement of "Specificity" (false-positive fraction), for all the possible cut-off values of the ICU assessment tools [17]. The area under this conventional binormal ROC curve is expressed as Az. The perfect predictive model, with no false-positive or false-negative results, would have an Az of 1 and, on the contrary, a model with an Az almost equal to 0.5 would not discriminate between ICU survivors and non-survivors. ROC analysis and the statistical significance of the differences in the Az were calculated using the ROCKIT 0.9b software (Department of Radiology, University of Chicago, Chicago, IL, USA). Differences in ROC pairs between the models were assessed implementing the techniques presented by Hanley and McNeil [18].

## Results

**Figure 1:**
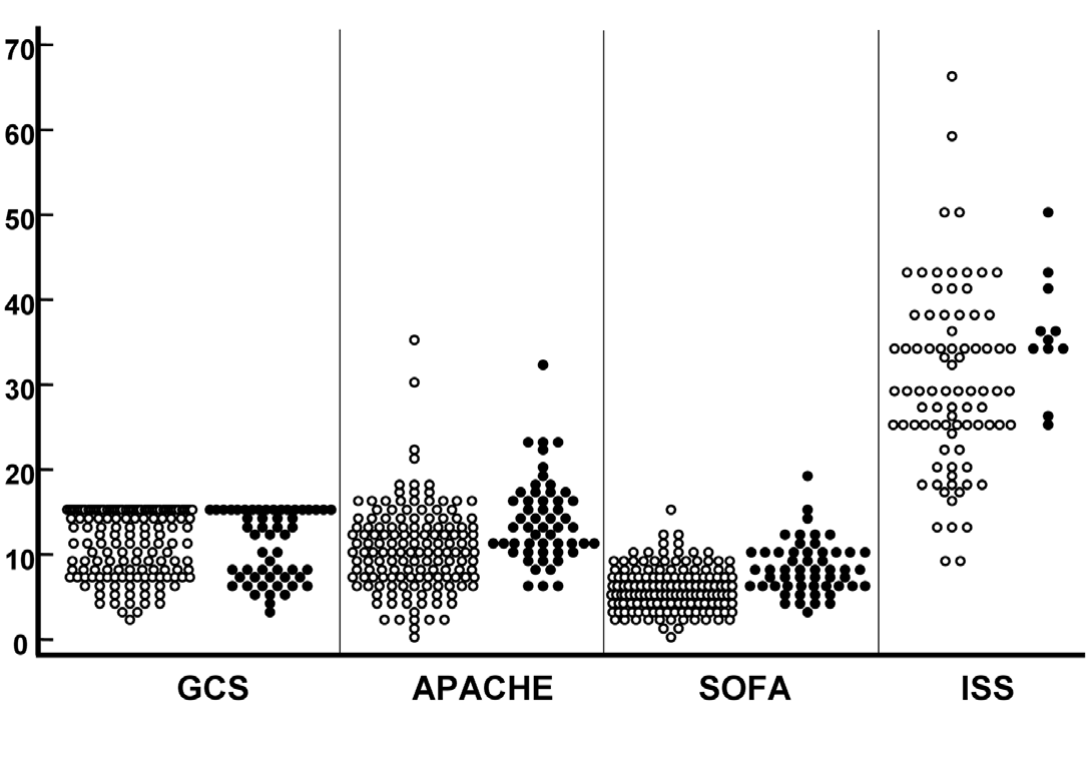
**Scores of critical care assessment tools in the study patients GCS, APACHE II, SOFA and ISS in survivors (open circles) and non-survivors (closed circles).** Note the considerable overlap between survivors and non-survivors.

Differences between survivors and non-survivors were seen in the APACHE II and the SOFA score (t-test F = 19.179, p < 0.001 and K-W H = 2.661, p < 0.001, respectively) whilst there were no significant differences between survivors and non-survivors in the GCS and the ISS (K-W H = 0.1236, p = 0.720 and t-test F = 3.296, p = 0.073, respectively). However, as shown in Figure 1, considerable overlap was noted in the mentioned above variables between survivors and non-survivors.

The three mining models (DTM, NNM, and LRM) were created including the GCS, ISS, APACHE, and SOFA as variables.

**Figure 2:**
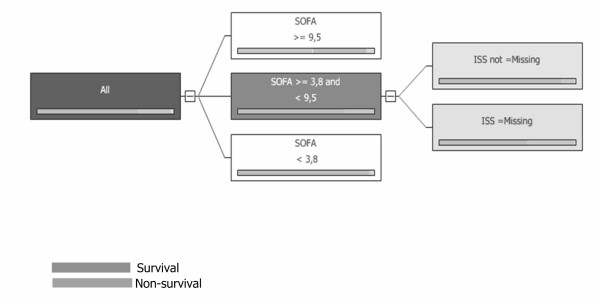
The DTM results.

**Figure 3:**
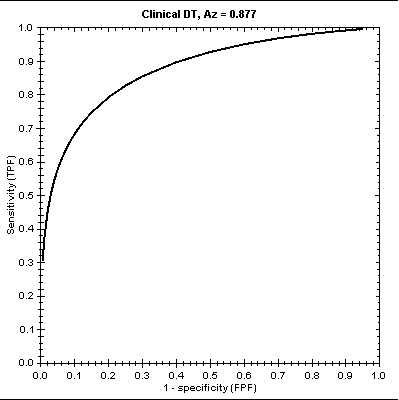
The ROC curve and the Az score in DTM.

For the DTM [see Additional file 1] we indentified specific and subcases. The limits [3.8<SOFA, 3.8 ≤ SOFA< 9.5, with two sub-cases IIS missing/IIS not missing (for patients with no trauma, or with trauma, respectively), and SOFA = 9.5] for each subset of the train set are illustrated in Figure 2. The Hosmer-Lemeshow statistic had a chi square value of 3.830 for this model (p = 0.147). The ROC curve for this DTM is plotted in Figure 3 whilst the estimated Az score ± SE was 0.8773 with 95% Confidence Interval (.7878, .9363).

**Figure 4:**
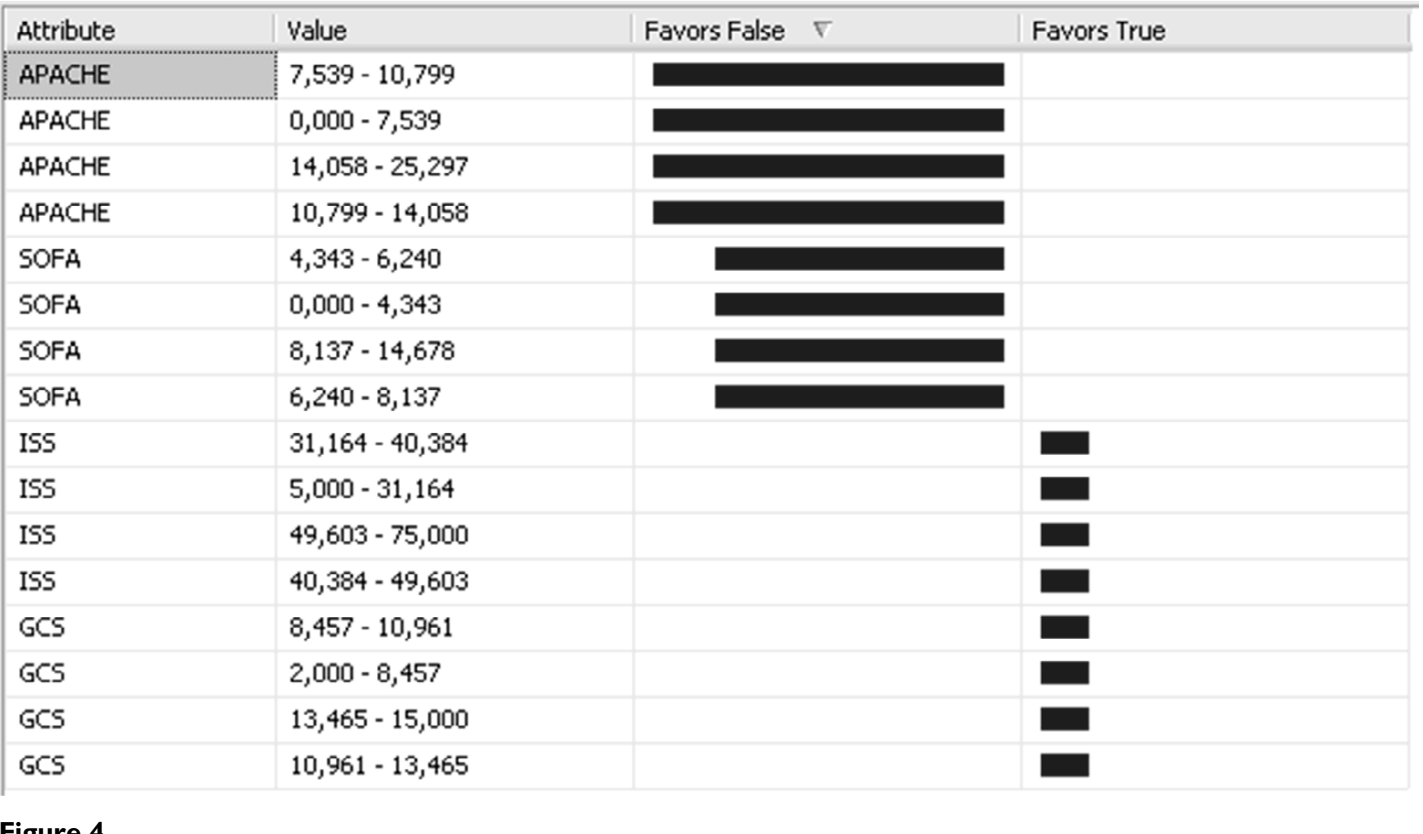
The NNM results.

**Figure 5:**
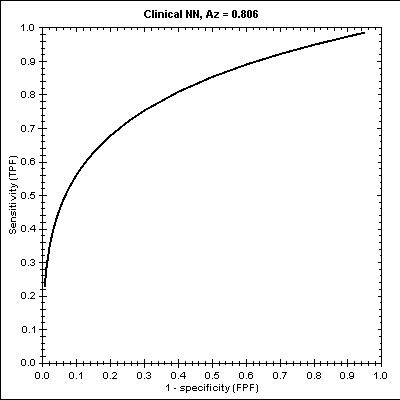
The ROC curve and the Az score in NNM.

The NNM [see Additional file 2] was structured upon three layers: the input layer, the hidden layer, and the output layer. Figure 4 illustrates the impact of each input variable, the identified limits (0.000 ≤ APACHE ≤ 14.058, 0.000 ≤ SOFA ≤ 14.678) and the False and True probability concerning *Survival*. The Hosmer-Lemeshow statistic had a chi square value of 10.451 for this model (p = 0.238). The ROC curve for this NNM is plotted in Figure 5 whilst the estimated Az score ± SE was 0.8061± 0.0427 with 95% Confidence Interval (.7119, .8787).

**Figure 6:**
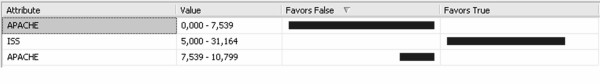
The LRM results.

**Figure 7:**
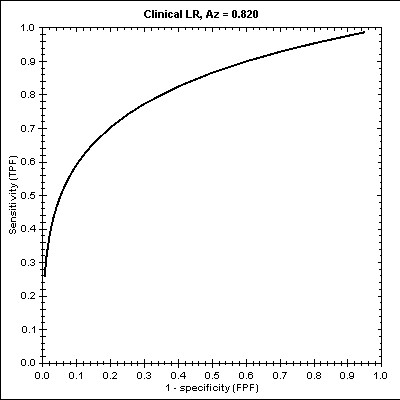
The ROC curve and the Az score in LRM.

Finally, we structured an LRM [see Additional file 3] that was based on the above NNM by removing the hidden layer. Figure 6 illustrates the LRM the impact per variable as well as the indentified limits (0.000 = APACHE<7.539, 7.539 = APACHE = 10.799) concerning *Survival*. The Hosmer-Lemeshow statistic had a chi square value of 9.858 for this model (p = 0.275). The ROC curve for the LRM is plotted in Figure 7 whilst the estimated Az score ± SE was 0.8204± 0.0376 with 95% Confidence Interval (.7276, .8903).

The differences in area under the ROC curves for the different diagnostic models had values of 0.0712, 0.0569 and 0.0143 for the DTM-NNM, DTM-LRM and LRM-NNM combinations respectively.

## Discussion

Through the use of data mining models, research in healthcare area is discovering new approaches and patterns of behavior that previously went unnoticed. The first step toward building a new approach is, of course, to gather and combine data whilst the whole process usually depends upon the gathered data, the problem to solve and the tools available.

As mentioned before, the present study proposes a prototype meta-level predicting approach concerning Intensive Care Unit (ICU) survival. The key characteristic of this study is that it "drags the whole process one step forward", at a meta-level, considering classic scoring systems e.g. APACHE II as variables considering all the operations and indicators of the process as equivalent.

For the DTM, the data were first split recursively into subsets so that each subset contained rather homogeneous states of the predictable variable (survival/or non-survival). Following this split, all input attributes were evaluated for their impact on the predictable attribute and the DTM performed. Finally, the behavior of the embedded algorithm was controlled by a set of input parameters (score method, split method, minimum support etc.).

Our findings indicate that the structured DTM is delimited at SOFA values of 3.8 and 9.5. When the SOFA variable value was lower than 3.8, the probability of survival was increased (a situation of almost certain survival). When it was between these two bounds (3.8 and 9.5), the prediction capacity was limited whilst we identified two sub-cases regarding the ISS: with (in case of trauma) or without an ISS score (in case of no trauma). In the sub-case with no trauma, the probability of survival was higher compared to sub-cases with trauma. Finally, when the SOFA variable value was higher than 9.5, the probability of non-survival was increased. By structuring the ROC curve for this model, we found that the Az score was 0.877.

For the NNM, as mentioned before, we used three types of nodes: input (SOFA, GCS, ISS, and APACHE), one hidden, and output (True/False for Survival/Non survival). Our findings indicate that the most significant variable with increased predictive capacity is first the APACHE (0.000 ≤ APACHE ≤ 14.058) and second the SOFA (0.000 ≤ SOFA ≤ 14.678) score. By structuring the ROC curve for this model, we found that the Az score is ~0.806. Thus with little variance we can conclude that like the DTM, the NNM succeeded to detect correctly the nonlinear relationships among input attributes (SOFA, GCS, ISS, and APACHE) as well as of *Survival*.

On the other hand, the LRM is a prediction model that is better suited to situations with the predictable variable having two possible states (survival/non-survival). Our findings have also indicated that the APACHE variable is a crucial indicator (especially in the region of 0.000 ≤ APACHE<7.539) for the False outcome and the ISS for the True outcome. We estimated that the model achieved an Az score of 0.820.

Available prediction models fail to integrate the required accuracy for prognosis of patients with similar severity of illness [6]. These models do not adequately support the generation of risk classifications. Consequently, the main issue in such predicting processes is the appropriate selection of the DTM as initiated in data mining processes.

The overall results for our data set are quite satisfactory as far as misclassification rates are concerned; misclassification rates lower than 33% seem to be acceptable in many circumstances. However, the detailed review of the results presented suggests that the performance of the DTM was superior to that of the NNM and the LRM when applied to the given data set, with respect to sensitivity and specificity (which are of great clinical relevance). The Az for the DTM showed better discriminative capabilities (accuracy of predicting whether a given subject would survive or not) as compared to NNM and LRM (only the DTM achieved a near optimal Az score).

Although there was a statistically significant difference in discrimination as measured by ROC curve analysis in favor of the DTM, the clinical meaning of this difference is not clear. A prediction model cannot be both perfectly reliable (i.e. calibrated) and perfectly discriminatory. We used the default parameters of the inner algorithms to configure each predictive model whilst for the DTM, due to the small number of subjects in the training set; we adjusted the minimum support parameter value equal to five.

On the other hand it should be noted that the present study has limitations that need to be taken into account when considering the study and its contributions. Sample size was small and we also used admission GCS, an assessment tool that suffers inherently from the patients' condition (for example by the patients' hemodynamic status as in cardiac trauma patients). This is a known shortcoming of the GCS score but it is a time-proven tool and has been used in a vast array of retrospective studies. With the available data only ICU mortality could be assessed. Although it is hospital mortality that may help to assess societal impact as an outcome of ICU hospitalization, ICU mortality is very useful in studies that assess the relationships of various predictors vis-à-vis outcome, as well as in studies that assess cause and effect relationships[19]. In this study APACHE II was used for trauma patients, a population for which it may not be best-suited. Nevertheless, its use was mandated by its widespread use. SOFA and APACHE II show interdependency, which may give suspect effects particularly in logistic regression. However, the aim of the study was to include all available information (regardless of such interactions) and look for "hidden patterns" in these parameters. The study of the clinical relevance using alternative methods (split method, minimum support etc.) in the tree based model would also be interesting, but it requires a larger clinical database. Finally, this was a retrospective study performed at a single tertiary-care medical center; thus generalization of findings is limited.

We must note that this study is not an additional tool for comparing directly ICU prognostic indicators. This study expands existing works in order to introduce a meta-level predicting approach concerning ICU survival. The approach and the findings should be used to support predicting processes in the future, to further evaluate they usefulness and completeness. They should be reviewed carefully by other research groups, using large data sets to provide essential feedback.

## Conclusion

Daily, in ICUs huge amounts of data are collected and reviewed, in routine clinical practice; decision support tools that use such data may be controversial. The predicting processes of ICU survival may go "one step forward", by using classic composite assessment indicators e.g. APACHE II as variables.

## Competing interests

The authors declare that they have no competing interests.

## Authors' contributions

LGG conceived the paper, carried out the mathematical analysis, and drafted the paper, FS carried out the mathematical analysis, II conceived the paper, carried out the mathematical analysis, and drafted the paper, KS performed the clinical measurements and collected data, ID performed the clinical measurements, collected data and drafted the manuscript. All authors read and approved the final manuscript.

## Pre-publication history

The pre-publication history for this paper can be accessed here:



## Supplementary Material

Additional file 1 OC – Test out for DTM. The additional file 1 presents the test out file (numerical results) of the DTM. Click here for file

Additional file 2 ROC – Test out for NNM. The additional file 2 presents the test out file (numerical results) of the NNM. Click here for file

Additional file 3 ROC – Test out for LRM. The additional file 2 presents the test out file (numerical results) of the LRM. Click here for file
